# The pathogenicity of novel *GUCY2D* mutations in Leber congenital amaurosis 1 assessed by HPLC-MS/MS

**DOI:** 10.1371/journal.pone.0231115

**Published:** 2020-04-07

**Authors:** Xue Feng, Tianying Wei, Junhui Sun, Yuqin Luo, Yanan Huo, Ping Yu, Jiao Chen, Xiaoming Wei, Ming Qi, Yinghui Ye

**Affiliations:** 1 Department of Cell Biology and Medical Genetics, School of Medicine Zhejiang University, Hangzhou, Zhejiang, China; 2 Department of Reproductive Genetics, Women’s Hospital, School of Medicine Zhejiang University, Hangzhou, Zhejiang, China; 3 Department of Eye Center, The Second Affiliated Hospital of School of Medicine Zhejiang University, Hangzhou, Zhejiang, China; 4 BGI-Wuhan, Wuhan, China; 5 Department of Reproductive Endocrinology, Key Laboratory of Reproductive Genetics, Ministry of Education, and Women’s Reproductive Health Laboratory of Zhejiang Province, Women’s Hospital School of Medicine Zhejiang University, Hangzhou, Zhejiang, China; Carl von Ossietzky Universitat Oldenburg, GERMANY

## Abstract

Leber congenital amaurosis (LCA) is a group of severe congenital retinal diseases. Variants in the guanylate cyclase 2D gene (*GUCY2D*), which encodes guanylate cyclase 1 (ROS-GC1), are associated with LCA1 and account for 6%–21% of all LCA cases. In this study, one family with LCA1 was recruited from China. A combination of next generation sequencing and Sanger sequencing was used to screen for disease-causing mutations. We found three novel mutations (c.139delC, p.Ala49Profs*36; c.835G>A, p.Asp279Asn and c.2783G>A, p.Gly928Glu) in the *GUCY2D* gene. Proband III-2 carries mutations c.139delC and c.2783G>A, which are inherited from the heterozygous mutation carriers, II-2 (c.139delC) and II-3 (c.2783G>A) that possess c.139delC and c.2783G>A. Additionally, II-8 carries heterozygous mutation c.835G>A. Sanger sequencing was used to confirm the presence of the three novel mutations in other family members. Mutation c.139delC results in a truncated protein. Mutations c.835G>A and c.2783G>A significantly reduce the catalytic activity of ROS-GC1. Our findings highlight the gene variants range of LCA. Moreover, HPLC-coupled tandem mass spectrometry (HPLC-MS/MS) was used to analyze the concentration of 3',5'-cyclic guanosine monophosphate (cGMP), suggesting that HPLC-MS/MS is an effective alternative method to evaluate the catalytic activity of wild-type and mutant ROS-GC1.

## Introduction

Leber congenital amaurosis (LCA), accounts for at least 5% of all inherited retinal dystrophies, and is the earliest and most severe form of all inherited retinal dystrophies [1]. LCA is generally inherited in an autosomal recessive manner [2–4] and characterized by genetic and phenotypic heterogeneity. Currently, mutations in 20 genes are associated with LCA [5]. The distribution of pathogenic genes vary considerably among different populations; however, guanylate cyclase 2D (*GUCY2D*) is a prevalent LCA gene because mutations to *GUCY2D* account for 6%-12% of all LCA (LCA1) cases [4, 5].

Guanylate cyclase 1 (ROS-GC1), encoded by *GUCY2D*, is expressed in rod and cone cells of vertebrae retina and catalyzes the synthesis of 3',5'-cyclic guanosine monophosphate (cGMP). ROS-GC1 is negatively controlled by a Ca^2+^ feedback loop because a reduction in Ca^2+^ concentration causes an increase in ROS-GC1 activity. However, the effects of the Ca^2+^ concentration on ROS-GC1 activity are indirect because this feedback loop is regulated by small Ca^2+^-binding guanylate cyclase-activating proteins (GCAPs), which interact with overlapping binding sites within the juxtamembrane domain (JMD), kinase homology domain (KHD) and dimerization domain (DD) of ROS-GC1. In addition to these latter domains, ROS-GC1 also possesses a leader sequence (LS), an extracellular domain (ECD), a transmembrane domain (TM) and a cyclase catalytic domain (CCD) [6]. Currently, 127 *GUCY2D* point mutations in different domains of ROS-GC1 cause protein dysfunction and subsequently result in LCA1 [5].

In the present study, targeted-next generation sequencing (NGS) was performed to screen 222 genes that are responsible for 24 kinds of ophthalmic genetic diseases in the proband. After confirming the results by Sanger sequencing, the identity of three novel variants of the *GUCY2D* gene were found, c.139delC (p.Ala49Profs*36), c.835G>A (p.Asp279Asn) and c.2783G>A (p.Gly928Glu). Mutations c.139delC and c.2783G>A were carried by proband III-2, and heterozygous mutation c.835G>A was carried by II-2. This study probes the pathogenesis of LCA1 and addresses the effects of mutations on ROS-GC1 activity. The three novel mutations may be suitable for LCA1 screening, and HPLC-MS/MS represents a convenient and effective method for cGMP quantification.

## Materials and methods

### Ethics approval and consent to participate

All procedures performed were in accordance with the Declaration of Helsinki and approved by the ethical standards of the Institutional Review Board, Zhejiang University. Written informed consent was obtained from all individuals who participated in this study and the parent of children under 16.

### LCA patients

A family with a 5-year-old proband (III-2) and two suspected patients (III-1 and III-3) was recruited for this study. The proband was diagnosed with LCA at Guangdong Zhongshan Hospital, Shanghai General Hospital and the Second Affiliated Hospital of the School of Medicine, Zhejiang University. The pedigree was constructed for the proband based on information provided by the guardians.

### DNA isolation and qualification

Total genomic DNA was extracted using the Relax Gene Blood DNA System (Tiangen, Beijing, China) following the manufacturer’s instructions. All DNA was dissolved in sterilized double-distilled water and kept at –20 °C until assayed.

One percent agarose gels were used to monitor DNA degradation and contamination. All DNA samples were examined for protein contamination (as indicated by the A_260_/A_280_ ratio) and reagent contamination (indicated by the A_260_/A_230_ ratio) with a NanoDrop ND 1000 spectrophotometer (NanoDrop, Wilmington, DE, USA).

### Targeted-next generation sequencing (NGS)

DNA samples obtained from the proband were sequenced using microarray-based targeted-NGS. A customized Agilent SureSelect All Exome Kit (Agilent Technologies, CA, USA) was designed to capture 3093 exons (including 100 bp regions that flanked the exons) from 222 genes known to be associated with common genetic diseases, including retinitis pigmentosa, Waardenburg syndrome, X-linked juvenile retinoschisis, crystalline retinitis pigmentosa, albinism, LCA, Bardet Biedl syndrome and cone-rod dystrophy (Additional Table 1). The procedure for the preparation of the libraries was consistent with standard protocols published previously [7]. The data of targeted-NGS assay was analyzed by Illumina basecalling Software 1.7.

**Table 1:** Primers for mutation screening.

Name	Sequence	Length (bp)
***GUCY2D*-exon1-F**	GAAGCCAGGACAGATCCCAC	322
***GUCY2D*-exon1-R**	GGAGGCGTCAGGGGTCA	
***GUCY2D*-exon2-1-F**	GCCCCAGTTAGTCTTCCCAG	550
***GUCY2D*-exon2-1-R**	AGGGTTCACCGGACCCAC	
***GUCY2D*-exon2-2-F**	TGCTGCCCGAGCCTTG	549
***GUCY2D*-exon2-2-R**	TCCTCGCCACCCAGCA	
***GUCY2D*-exon3-F**	CAGGTAGGCTCCCTTGCAG	496
***GUCY2D*-exon3-R**	AGAGCTGCCAGTGGTTCTTT	
***GUCY2D*-exon4-F**	AACAGTGGATACCCTGGGC	550
***GUCY2D*-exon4-R**	GGGCATCGAAGACGGATTAC	
***GUCY2D*-exon5-F**	ATTCCCAGCCTCTCCCCTTT	387
***GUCY2D*-exon5-R**	ACTCTACCAGCCCACCAGAA	
***GUCY2D*-exon6-F**	TTCTGGTGGGCTGGTAGAGT	464
***GUCY2D*-exon6-R**	CCCGCCAGGAAAGTAGTCAG	
***GUCY2D*-exon7-F**	CCTGACTACTTTCCTGGCGG	477
***GUCY2D*-exon7-R**	TCCCTAGATCCTGTCTGGGC	
***GUCY2D*-exon8-F**	ATGGCTGTGAAGTGGATGGG	423
***GUCY2D*-exon8-R**	ATCCTCCCTCACATCTGCCT	
***GUCY2D*-exon9-F**	GAGAGCCCCCGTACATACCT	487
***GUCY2D*-exon9-R**	CGCCTCGATGGTGCAGATAC	
***GUCY2D*-exon10-F**	ATCAAGGTGTGTGTCTGGGG	476
***GUCY2D*-exon10-R**	CAGCTCAGGTACAAGTCCCG	
***GUCY2D*-exon11-F**	GGCCTATTTGCCAGGCTTTC	476
***GUCY2D*-exon11-R**	ACCTGCAGATGCCAGCTTT	
***GUCY2D*-exon12-F**	CTAGCAACCCCCTTCCACAC	402
***GUCY2D*-exon12-R**	AGCTGTCTCAGGTTGCTGAC	
***GUCY2D*-exon13-F**	ACGTGCCTCCTAATCGTGTC	505
***GUCY2D*-exon13-R**	GCTCAAAGTACTCGGGCTCC	
***GUCY2D*-exon14-F**	GCTGCTTACACAGATGCTGC	440
***GUCY2D*-exon14-R**	TAAGGGACAGGAGGTCTGGG	
***GUCY2D*-exon15-F**	CAGCTCAGTCCTTCCACTAGC	468
***GUCY2D*-exon15-R**	CGCACCCATTATCTCCACCA	
***GUCY2D*-exon16-17-F**	AGAGGATGCACTTAACAAGGCT	538
***GUCY2D*-exon16-17-R**	ATCTCGAGTCTGCGTGGAAC	
***GUCY2D*-exon18-F**	CCCTGTCCTGAGGCACC	365
***GUCY2D*-exon18-R**	CTCAGGGAAGGGGAATGGG	
***GUCY2D*-exon19-F**	CGAGGGACCCCTGCCT	393
***GUCY2D*-exon19-R**	ATTCCTGCAATGGCTGCTTC	
***GUCY2D*-exon20-F**	TAGCTGGCAGAGCAGTGATG	487
***GUCY2D*-exon20-R**	ACTTCCCCTCTTCAGGCCAT	

### Mutation validation by Sanger sequencing

Sanger sequencing was used to validate candidate variants identified by NGS. Primers of the *GUCY2D* gene (NG_009092.1) used in Sanger sequencing were designed by Primer-BLAST (http://www.ncbi.nlm.nih.gov/tools/primer-blast/) and synthesized by Sangon Biotech (Shanghai, China) (Table 1). All amplifications were examined by electrophoresis using 2% agarose gels and sequenced by BioSune Biotechnology Co., Ltd. (Shanghai, China). The sequencing results were further compared and analyzed by Mutation Surveyor [8].

### Construction of wild-type (wt) and mutant human ROS-GC1 recombinant plasmids

The cDNA of human ROS-GC1 was obtained from Gene Copoeia (EX-Z0715-M98). Primers F and R containing *Xho*I and *Age*I restriction sites were used to amplify ROS-GC1 (Table 2). The pEGFP-N1 vector was digested with *Xho*I and *Age*I restriction enzymes. PCR amplification product was sub-cloned into pEGFP-N1 using ClonExpress (Vazyme, Nanjing, China) to give the recombinant plasmid pEGFP-GC1.

**Table 2:** Primers for construction of recombination plasmids.

Primer	Sequence	Restriction Enzyme
**F**	TCCGCTAGCGCTACCGGACTCAGATctcgagATGACCGCCTGCGCCCGCCGAGCGGGTGGGCTT	XhoI
**R**	CTCGCCCTTGCTCACCATGGTGGCGaccggtGGTGAAGAGAACTGGCCCGGCCGCGCC	AgeI
**f-835**	GGCTCCCTGGTCTTCCTGCCCTTCaACACGATCCACTACGCCTTGTCCCCA	
**r-835**	TGGGGACAAGGCGTAGTGGATCGTGTtGAAGGGCAGGAAGACCAGGGAGCC	
**R-BamHI**	GGCGGAGAGGAAGGAGCCCCGGGCAggatccAGCATGTATGTGGCAAAAAGCCGGT	BamHI
**F-BamHI**	ACCGGCTTTTTGCCACATACATGCTggatccTGCCCGGGGCTCCTTCCTCTCCGCC	BamHI
**f-2783**	CTACAAGGTGGAGACAATAGaGGACGCCTATATGGTGGCC	
**r-2783**	GGCCACCATATAGGCGTCCtCTATTGTCTCCACCTTGTAG	

Site-directed mutagenesis PCR was used to construct the ROS-GC1 mutants. Primers used in site-directed mutagenesis PCR are shown in Table 2. Each mutant was achieved by two-step PCRs using pEGFP-GC1 as the template. For c.835G>A (p.Asp279Asn) two pairs of primers, F and r-835 and R-BamHI and f-835, were used in the first PCR step. Primers F and R-BamHI were used in the second step. For c.2783G>A (p.Gly928Glu), primers F-BamHI and r-2783 and R and f-2783 were used in the first PCR step. Primers F-BamHI and R were used in the second step. For each mutation, amplification products in the first step were cleaned (Axygen, CA, USA), mixed and used as the template in the second PCR reaction. All final PCR amplifications were ligated into the digested pEGFP-N1 vector using ClonExpress.

Recombinant plasmids pEGFP-GC1, pEGFP-Asp279Asn and pEGFP-Gly928Glu were transformed into Escherichia coli DH5α cells. DNA was prepared by using a plasmid DNA purification kit from Macherey-Nagel following the manufacturer’s instructions. Sanger sequencing was used to verify sequences.

### Cellular localization of wt and mutant ROS-GC1 recombinant plasmids

pEGFP-N1, pEGFP-GC1, pEGFP-Asp279Asn and pEGFP-Gly928Glu were transfected into HeLa cells using the PolyJet Reagent (SigmaGen, MD, USA). After 36 h, cells were washed and fixed in 4% paraformaldehyde. The antibody anti-Na^+^/K^+^-ATPase (1:100, HuaAn Biotechnology Co., Ltd., Hangzhou, China) was used to identify the plasma membrane of HeLa cells. Details about the process have been described previously [9]. In HeLa cells, the localization of pEGFP-N1, pEGFP-GC1, pEGFP-Asp279Asn and pEGFP-Gly928Glu was acquired by observing EGFP at 488nm excitation wavelength using a Nikon A1R.

### Western blotting

Proteins from transfected HeLa cells were electrophoresed on a 12% sodium dodecyl sulfate-polyacrylamide gel (SDS-PAGE) and transferred to polyvinylidene fluoride (PVDF) membranes (Millipore, MA, USA), and then incubated with the primary antibodies, anti-GFP (1:1000, Proteintech, Wuhan, China) or anti-glyceraldehyde 3 phosphate dehydrogenase (GAPDH) (1:5000, Abcam, Cambridge, UK) overnight at 4 °C. The PVDF membranes were washed with PBS and incubated with fluorescent secondary antibodies (1:1000, Abbkine, Wuhan, China) for 2 h at room temperature. The protein bands were visualized using an Odyssey Imager (Li-Cor Biosciences, NE, USA). GAPDH was used as the standard.

### Validation of cGMP quantitation by HPLC-MS/MS

HeLa cells were transfected with pEGFP-N1, pEGFP-GC1, pEGFP-Asp279Asn and pEGFP-Gly928Glu. After 36 h, cells were collected from 100 mm plates and washed three times with PBS. The supernatant was removed carefully and 300 μL of ice-cold extraction medium (acetonitrile/methanol/water, 2/2/1 v/v/v) was added to each tube. Twenty-five ng/mL Tenofovir (TNF) was added as the internal standard. After dissolving, the sample was frozen immediately in liquid nitrogen for 30 s to terminate cGMP metabolism, and this was followed by incubation in a 37 °C water bath for 60 s. After repeating 6 times, samples were heated at 98 °C for 20 min. Samples were cooled on ice and centrifuged at 20,000 × *g* at 4 °C for 10 min. The supernatant was transferred into a new tube and the non-dissolved residue was extracted two more times with 400-μL extraction medium. After evaporation, residues were collected and dissolved in water for further analysis.

The cGMP concentrations were analyzed via HPLC-MS/MS. The samples were applied to an HPLC utilizing an ACQUITY CSH-C18 column (1.7 μm, 2.1 × 100 mm column, Waters, Ireland). The binary pump system supplied two eluents for chromatographic analysis, eluent A (10 mM formic acid) and eluent B (acetonitrile). The flow rate was 0.3 mL/min. Analyte detection was conducted on a sensitive triple quadrupole mass spectrometer (Waters TQ-XS, USA). Nitrogen was used as the collision gas. One-hundred ng/mL cGMP (G7504, Sigma, Germany) was used as the standard. All processes were referenced to the method described previously [10]. cGMP concentrations presented as means ± SEM are based on three repeated measurements. *P*-values were calculated by means of the independent sample T-test.

### Bioinformatics analysis

All sequences were analyzed by Mutation Surveyor software and aligned to the NCBI nucleotide sequence of *GUCY2D* (NG_009092.1). The pathogenicity of mutations was evaluated using the *in silico* predictors SIFT (http://sift.jcvi.org/), PolyPhen-2 (http://genetics.bwh.harvard.edu/pph2/) and Mutation Taster (http://www.mutationtaster.org/). Computational modeling of the mutant ROS-GC1 by Chimera (PDB ID: 1AWL) was carried out to study the effect of the p.Gly928Glu mutation on the three-dimensional (3D) structure of ROS-GC1.

## Results

### Clinical description of the proband

The proband (III-2) was a five-year-old girl who showed the disorder visually but could perceive light in a dark room. The proband displayed the classic signs of oculo-digital symptoms. Ophthalmological examination revealed pendular nystagmus, roving eye movements, macular “colobomas” and optic disc abnormalities. Additionally, dim retina and salt-and-pepper pigmentation were found in the fundus. The results of flash visual evoked potential (VEP) showed that the latent period of binocular P-waves was severely prolonged and the amplitudes were severely reduced. The electroretinogram (ERG) results showed that the latent period and the amplitude of binocular scotopia rod response b-wave, mixed response a-wave and mixed response b-wave were flat. With an attenuated photopic 30-Hz flicker, the full-field ERG (ffERG) showed a worsening delay of the cone-dependent response. The latent period and amplitude of the binocular photopic cone response a-wave and b-wave were flat. Clinical examination of the parents confirmed that neither was affected.

### *GUCY2D* mutation analysis

**Fig 1:**
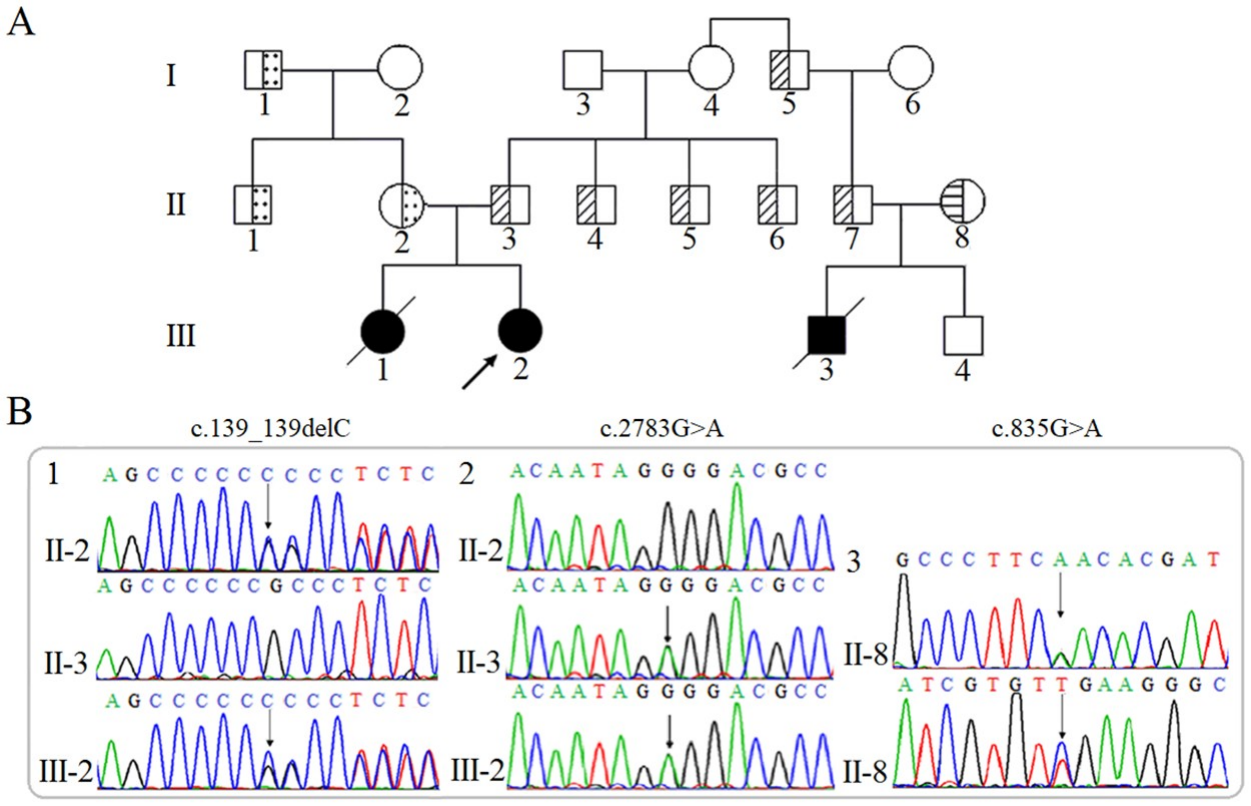
Brief introduction to the *GUCY2D* mutations in the LCA1 family. (A) Pedigree of the LCA1 family. ↗ represents the proband, □ represents the normal male, ○ represents the normal female and crosses represent the deceased subject. ● and ■ denote patients, whereas the half-shaded icons denote the mutation carriers as ⚅ = c.139delC (p.Ala49Profs*36), ▨ = c.2783G>A (p.Gly928Glu) and ▤ = c.835G>A (p.Asp279Asn). (B) 1: The sequencing results of the family show the mutation c.139delC, indicated by the arrows. 2: The sequencing results of the family show the mutation c.2783G>A, indicated by the arrows. 3: The sequencing result of the family member II-8 with mutation c.835G>A; forward sequencing result (top) and reverse sequencing result (bottom).

In order to discover the pathogenesis of the proband, targeted-NGS was performed using Agilent SureSelect All Exome Kit with Hiseq2000 sequencer. After analyzing the sequencing results of targeted-NGS, two novel mutations, c.139delC and c.2783G>A, in the *GUCY2D* gene were identified in the proband (III-2). Sanger sequencing confirmed the presence of c.139delC and c.2783G>A in other family members. The sequencing results showed that II-2 and II-3 are heterozygous mutation carriers that possess c.139delC and c.2783G>A, respectively (Fig 1B1 and 1B2).

III-3 was described as an LCA1 patient (Fig 1A). Sanger sequencing was used to determine the *GUCY2D* gene sequences of the suspected sibling of the patient (III-4) and parents (II-7 and II-8). The results showed that II-7 is a c.2783G>A mutation carrier, and II-8 possesses another novel mutation c.835G>A (Fig 1B3). The sibling (III-4) possessed no *GUCY2D* mutation. In addition, all heterozygous mutation carriers showed no clinical symptoms.

### Prediction of the pathogenic effect of ROS-GC1 mutations

**Fig 2:**
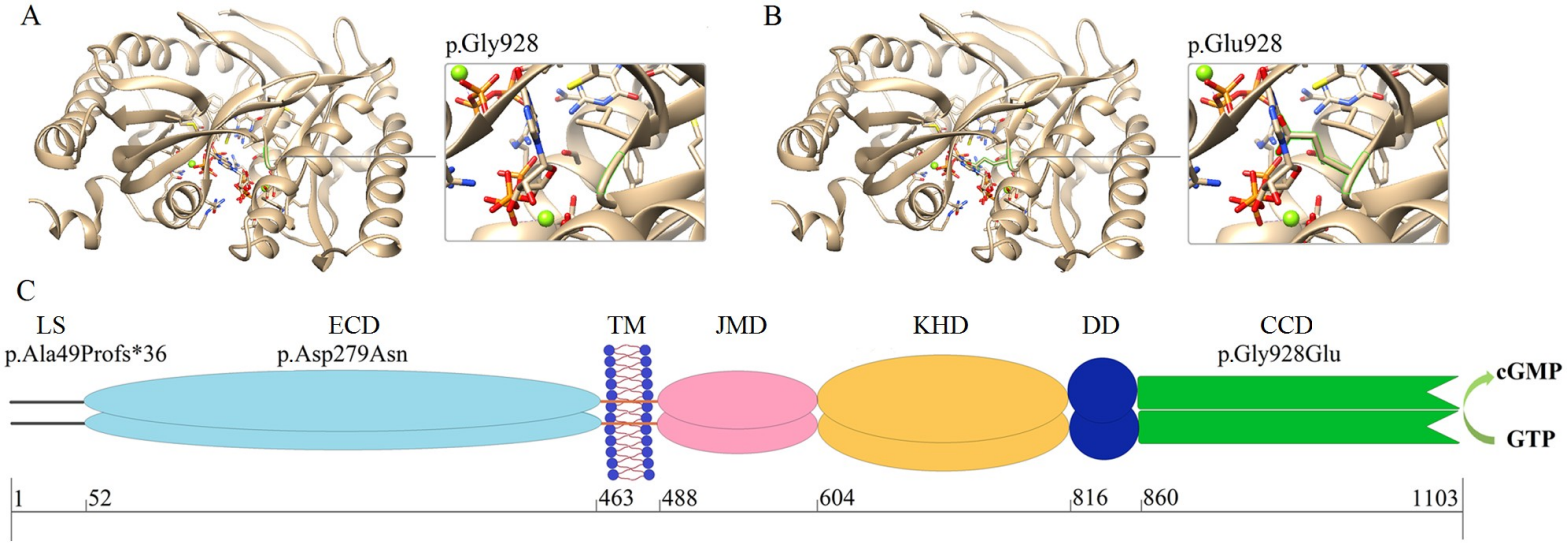
Protein domains and modeling analysis of the p.Gly928Glu mutation. (A) 3D-modeling analysis of Gly928. (B) 3D-modeling analysis of Glu928. The green line indicates the ROS-GC1 residue. (C) ROS-GC1 domains and location of p.Ala49Profs*36, p.Asp279Asn and p.Gly928Glu. LS: leader sequence; ECD: extracellular domain; TM: transmembrane domain; JMD: juxtamembrane domain; KHD: kinase homology domain; DD: dimerization domain; and CCD: cyclase catalytic domain.

p.Ala49Profs*36, p.Asp279Asn and p.Gly928Glu are located in the LS, ECD and CCD of ROS-GC1, respectively (Fig 2C). After analyzing the three mutations by SIFT, PolyPhen-2 and Mutation Taster, we propose that c.835G>A (SIFT: damaging, score: 0.01; PloyPhen-2: probably damaging, score: 1.00; Mutation Taster: disease-causing), c.2783G>A (SIFT: damaging, score: 0.00; PloyPhen-2: probably damaging, score: 1.00; Mutation Taster: disease causing) and c.139delC (Mutation Taster: disease-causing) are disease-causing mutations. Additionally, multiple sequence alignments showed that aspartic acid and glycine at positions 279 and 928 are highly conserved across species (S1 Fig), indicating that p.Asp279 and p.Gly928 play important roles in ROS-GC1 and that mutations to these two residues are likely to cause ROS-GC1 dysfunction.

The high-resolution 3D structure of full-length ROS-GC1 remains unsolved. The theoretical model (PDB ID: 1AWL), which contains 158 amino acids from 871 to 1028 was used to study the impact of c.2783G>A (p.Gly928Glu) on the 3D structure of ROS-GC1. 3D modeling analysis showed that Glu928 directly interacts with GTP (Fig 2) and this change may reduce the size of the catalytic core, leading to a change in the relative position of GTP and the catalytic core. Furthermore, this mutation likely hampers ligand binding and reduces the catalytic activity of ROS-GC1.

### Localization of wt and mutant ROS-GC1

**Fig 3:**
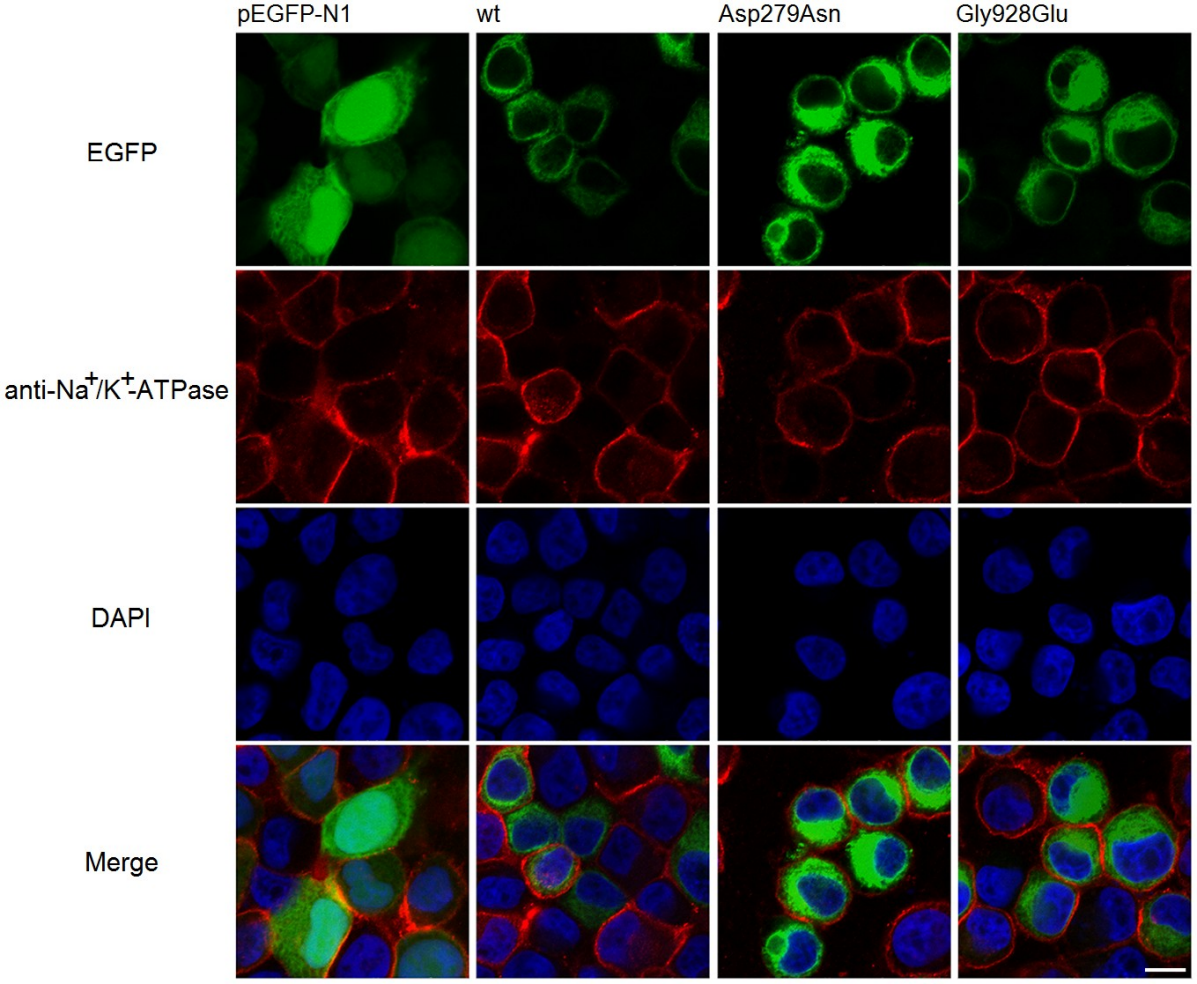
Cellular localization of wt and mutant ROS-GC1 in HeLa cells. Localization of ROS-GC1 wt and mutants were acquired by observing EGFP. Immunostaining from top to bottom: EGFP with 488nm excitation (top panels), anti-Na^+^/K^+^-ATPase (second panels) as the cell membrane marker, DAPI (third panels) as the nucleus marker, and overlay images (bottom panels). The scale bar is 10 μm.

ROS-GC1 is a member of the membrane guanylyl cyclase family, the correct localization of ROS-GC1 is critical for the synthesis of cGMP. In this study, immunofluorescence was used to confirm the localization of wt and mutant ROS-GC1. pEGFP-N1, pEGFP-GC1, pEGFP-Asp279Asn and pEGFP-Gly928Glu were transfected into HeLa cells after verification by Sanger sequencing (S2 Fig) and the localization was acquired by observing EGFP under the 488nm excitation. The localization of p.Asp279Asn and p.Gly928Glu was similar to the localization of the wt ROS-GC1. (Fig 3, top panels). The anti-Na^+^/K^+^-ATPase antibody was used as a specific marker of the plasma membrane (Fig 3, second panels).

### Catalytic features of wt and mutant ROS-GC1

**Fig 4:**
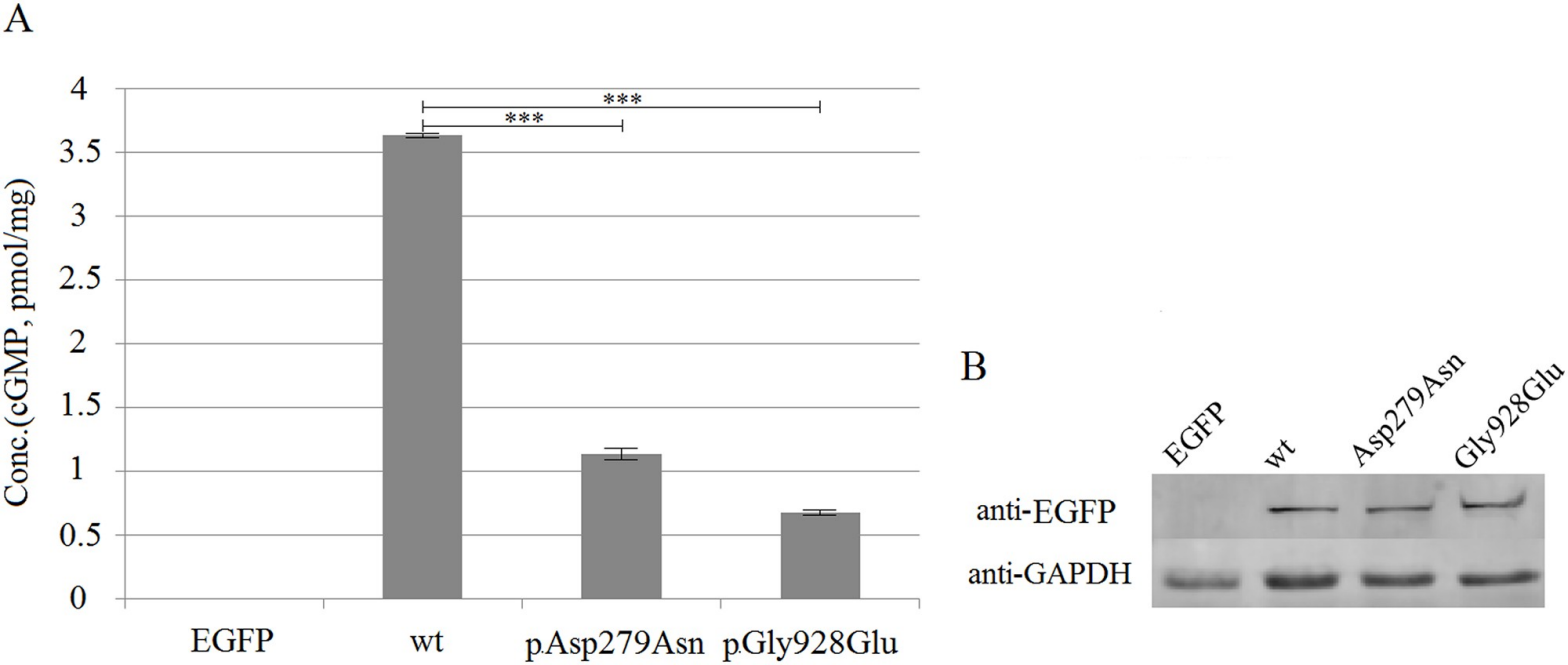
cGMP concentrations and ROS-GC1 expression levels in HeLa cells. (A) Transfected HeLa cells with pEGFP-N1, pEGFP-GC1, pEGFP-Asp279Asn and pEGFP-Gly928Glu. Cells were collected and used to estimate cGMP concentrations. *P*-values were calculated by means of an independent sample T-test. ***: *p* ≤ 0.001. (B) Western blot of HeLa cells expressing human EGFP, wt and mutant ROS-GC1.

HPLC-MS/MS was used to analyze cGMP concentrations in HeLa cells. The cGMP concentration in cells transfected with pEGFP-N1 was undetectable. In contrast, the cGMP concentration in cells transfected with pEGFP-GC1 was about 3.631 pmol/mL. Compared with wt, cells transfected with pEGFP-Asp279Asn or pEGFP-Gly928Glu showed significantly lower concentrations of cGMP (Fig 4A), implying that p.Asp279Asn and p.Gly928Glu significantly reduced the catalytic activity of ROS-GC1. In addition, p.Gly928Glu disrupted the catalytic activity of ROS-GC1 more severely than the other mutants, which is consistent with the bioinformatics analysis. Moreover, all missense mutations did not affect the expression level of ROS-GC1 (Fig 4B).

## Discussion

Twenty known genes associated with LCA have been identified. The large number of genes and exons and the lack of mutational hot spots make individual screening for disease-causing mutations by Sanger sequencing difficult and expensive. However, NGS technologies provide more convenient and effective strategies to screen for pathogenic mutations in genes [11–13]. In this study, a family with one LCA proband was analyzed. Targeted-NGS and Sanger sequencing were combined to identify three mutations (c.139delC, c.2783G>A and c.835G>A) in the *GUCY2D* gene in this pedigree, and all the mutations have yet to be documented by the 1000 Genomes Project. According to the description, there are two suspected patients in this pedigree, III-1 and III-3. However, both of them died at a very young age, thereby we only verified the *GUCY2D* gene mutation in II-7 and II-8. Additionally, in this pedigree all the heterozygous mutation carriers showed no clinical symptoms.

The novel disease-causing mutation c.139delC (p.Ala49Profs*36) carried by II-1 and putatively by II-2 and III-3 generates a truncated protein (83 amino acids), containing only a part of the LS (Fig 2C). As all the domains of ROS-GC1 are presumably absent, there is no enzymatic activity for this ROS-GC1 mutant and thus no cGMP production. Thus, the absence of sufficient levels of cGMP results in photoreceptor cell polarization, which finally leads to vision loss.

Multiple sequence alignments indicated that aspartic acid and glycine at positions 279 and 928 are highly conserved across species, indicating that these residues are important for protein function. This is consistent with previous observations that mutations located in the ECD decrease cGMP production and result in LCA1 [14]. The p.Asp279Asn mutation of this reported family decreased ROS-GC1 catalytic activity significantly; however, this mutation does not alter the cellular localization and the expression level of ROS-GC1. Mutant c.2783G>A (p.Gly928Glu) is located in the pocket like catalytic domain of ROS-GC1 [15]. Bioinformatics analysis was used to estimate the effects of p.Gly928Glu on the catalytic activity of ROS-GC1. In contrast to Gly928, Glu928 directly interacts with the substrate GTP. Alteration of the catalytic core pocket by the p.Gly928Glu mutation perturbs the catalytic activity of ROS-GC1. HPLC-MS/MS was used to further characterize the effect of the missense mutations on ROS-GC1 activity, and therefore provide additional experimental evidence of the effects of the mutations on ROS-GC1 activity. Results from HPLC-MS/MS showed that mutations p.Asp279Asn and p.Gly928Glu decreased ROS-GC1 activity significantly, especially p.Gly928Glu, consistent with the 3D-modeling and bioinformatics analysis by SIFT, PolyPhen-2 and Mutation Taster.

In this study, the endogenous concentration of cGMP in HeLa cells, which were transfected with pEGFP-N1, is too low to be measured. This phenomenon can be observed in other studies of *GUCY2D* mutations, such as HEK293 cells expressed mutant ROS-GC1 (A710V and P873R) showed undetectable cGMP concentrations, indicating that endogenous cGMP concentration exerted no contribution to cGMP levels. Although *GUCY2D* gene encodes a retina-specific protein, use other cell lines to investigate the localization and enzymatic activity of ROS-GC1 is feasible, for example, HEK293 cells were widely used to study the cellular localization of ROS-GC1 [9,10, 16,17,18]. It has been reported that ROS-GC1 was present in cell membranes and mainly co-localized with the endoplasmic reticulum in HEK293 cells [9,16]. However, in our study, ROS-GC1 exhibited endoplasmic reticulum localization, without obvious co-localization with plasma membrane marker in HeLa cells. The discrepancy may be explained by the different cells type and the different method used to detect the localization of ROS-GC1. It has been found that ROS-GC1 trafficking from reticulum to the plasma membrane was facilitated by co-expression of retinal degeneration 3 (RD3) protein [19]. Thus, impaired interaction between ROS-GC1 and RD3 cannot be ruled out.

## Conclusions

In this study, we found three novel mutations (c.139delC, c.2783G>A and c.835G>A) in the *GUCY2D* gene. Mutation c.139delC results in a truncated protein that lacks all functional domains of ROS-GC1. Although mutations c.835G>A and c.2783G>A showed normal protein expression levels and subcellular localization, both mutations significantly reduced the catalytic activity of ROS-GC1. As an alternative to radioisotope labeling assays, HPLC-MS/MS was used herein to analyze cGMP concentrations. Compared with traditional methods, HPLC-MS/MS is more convenient and is an effective alternative to evaluate the cGMP concentration in cells.

## Supporting information

S1 Fig Multiple sequence alignment of ROS-GC1 from different species. Results from multiple sequence alignment reveal that codon 279 and 928 where the mutations Asp279Asn and Gly928Glu occurred are located within a highly conserved region.
(TIF)

S2 Fig Sequencing results of wt and mutant *GUCY2D*. Sanger sequencing was used to verify the sequences of the recombinant plasmids pEGFP-GC1, pEGFP-Ala49Profs*36, pEGFP-Asp279Asn and pEGFP-Gly928Glu.
(TIF)

S1 Table Screening scope of the targeted-NGS in eye diseases. (DOCX)

S1 File (RAR)

S2 File (RAR)

S3 File (RAR)

S4 File (RAR)

S5 File (RAR)

S6 File (RAR)

S7 File (RAR)

S8 File (RAR)

S9 File (RAR)

S10 File (RAR)

S11 File (RAR)

## Abbreviations

CCD: cyclase catalytic domain
DD: dimerization domain
ECD: extracellular domain
GCAPs: guanylate cyclase-activating proteins
GUCY2D: guanylate cyclase 2D
HPLC-MS/MS: HPLC-coupled tandem mass-spectrometry
JMD: juxtamembrane domain
KHD: kinase homology domain
LCA: Leber congenital amaurosis
LS: leader sequence
NGS: next-generation sequencing
ROS-GC1: guanylate cyclase protein 1
TM: transmembrane domain
wt: wild type
