# Supplementary figures and images for: The pathogenicity of novel GUCY2D mutations in Leber congenital amaurosis 1 assessed by HPLC-MS/MS
Source: PLoS One. 2020 Apr 7;15(4):e0231115. doi: 10.1371/journal.pone.0231115 (PMC7138296; doi:10.1371/journal.pone.0231115)

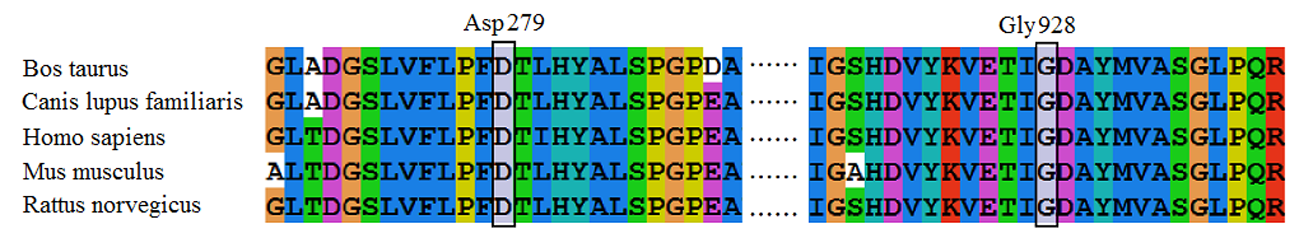

Supplement: S1 Fig — Results from multiple sequence alignment reveal that codon 279 and 928 where the mutations Asp279Asn and Gly928Glu occurred are located within a highly conserved region. (TIF) [file pone.0231115.s001.tif]

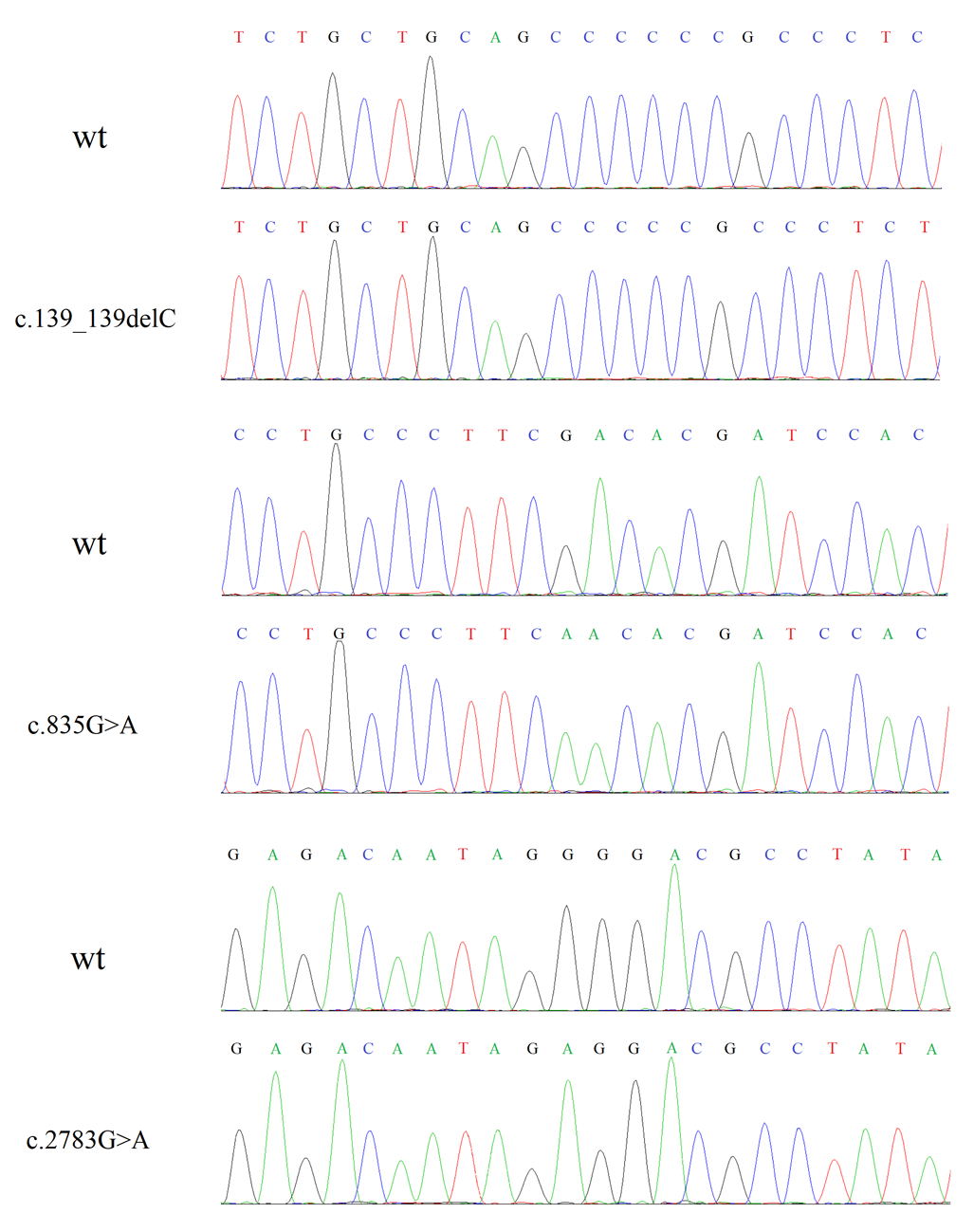

Supplement: S2 Fig — Sanger sequencing was used to verify the sequences of the recombinant plasmids pEGFP-GC1, pEGFP-Ala49Profs*36, pEGFP-Asp279Asn and pEGFP-Gly928Glu. (TIF) [file pone.0231115.s002.tif]
