# Supplementary material for: The pathogenicity of novel GUCY2D mutations in Leber congenital amaurosis 1 assessed by HPLC-MS/MS
Source: PLoS One. 2020 Apr 7;15(4):e0231115. doi: 10.1371/journal.pone.0231115 (PMC7138296; doi:10.1371/journal.pone.0231115)
Supplement: S1 Table — (DOCX) [file pone.0231115.s003.docx]

**S1 Table. Screening scope of the targeted-NGS in eye diseases.**

| **Disease name** | **Pathogenic** |
| --- | --- |
| **Retinitis pigmentosa (including autosomal dominant, autosomal recessive chain/X)** | *ABCA4*, *AIPL1*, *ARL6*, *BEST1*, *CA4*, *CERKL*, *C2orf71*, *CLRN1*, *DHDDS*, *NGA1*, *CNGB1*, *CRB1*, *CRX*, *EYS*, *FAM161A*, *FSCN2*, *GUCA1B*, *IDH3B*, *IMPDH1*, *IMPG2*, *KLHL*7, *LRAT*, *MAK*, *MERTK*, *NR2E3*, *NRL*, *PDE6A*, *PDE6B*, *PRCD*, *PDE6G*, *PROM1*, *PRPF3*, *PRPF31*, *PRPF6*, *PRPF8*, *PRPH2*, *RBP3*, *RDH12*, *RGR*, *RHO*, *RLBP1*, *ROM1*, *RP1*, *RP2*, *RP9*, *RPE65*, *RPGR*, *SAG*, *SEMA4A*, *SNRNP200, TOPORS*, *TULP1*, *USH2A*, *ZNF513* |
| **Waardenburg syndrome** | *EDNRB*, *MITF*, *PAX3*, *SNAI2*, *SOX10*, *EDN3* |
| **x-linked juvenile retinoschisis** | *RS1* |
| **Crystalline retinitis pigmentosa** | *CYP4V2* |
| **Bothnia retinal dystrophy** | *RLBP1* |
| **Best macular dystrophy** | *BEST1* |
| **Congenital aniridia** | *PAX6* |
| **Albinism (non syndromic)** | *GPR143*, *MITF*, *OCA2*, *TYR*, *OCA2*, *TYRP1*, *SLC45A2* |
| **Leber congenital amaurosis** | *GUCY2D*, *RPE65*, *SPATA7*, *AIPL1*, *LCA5*, *RPGRIP1*, *CRX*, *CRB1*, *NMNAT1*, *CEP290*, *IMPDH1*, *RD3*, *RDH12*, *LRAT*, *TULP1*, *KCNJ13* |
| **Bardet Biedl syndrome** | *BBS1*, *BBS2*, *ARL6*, *BBS4*, *BBS5*, *MKKS*, *BBS7*, *TTC8*, *BBS9*, *BBS10*, *TRIM32*, *BBS12*, *MKS1*, *INPP5E* |
| **Monochromasia** | *CNGA3*, *CNGB3*, *GNAT2*, *PDE6C* , *PDE6H* |
| **Protanopia anerythrochloropsia** | *OPN1LW*, *OPN1MW* |
| **Joubert syndrome** | *AHI1*, *ARL13B*, *CC2D2A*, *CEP290*, *KIF7*, *INPP5E*, *NPHP1*, *OFD1*, *RPGRIP1L*, *TMEM216*, *TMEM67* |
| **Senior-Loken syndrome** | *NPHP1*, *NPHP4*, *IQCB1*, *CEP290*, *SDCCAG8* |
| **Usher syndrome** | *CDH23*, *CLRN1*, *DFNB31*, *GPR98*, *MYO7A*, *PDZD7*, *PCDH15*, *USH2A*, *USH1C*, *USH1G* |
| **Age-related macular degeneration** | *ABCA4*, *TLR4*, *CST3*, *CFH*, *HTRA1*, *C3* |
| **Cone dystrophy** | *CACNA2D4*, *GUCA1A, KCNV2*, *PDE6H*, *PDE6C* |
| **Cone-rod dystrophy** | *ABCA4*, *ADAM9*, *AIPL1*, *BEST1*, *CACNA1F*, *CDHR1*, *CERKL*, *CRX*, *GUCA2A*, *GUCY2D*, *PITPNM3*, *PROM1*, *RAX2*, *RIMS1*, *RLBP1*, *RPGR*, *RPGRIP1*, *SEMA4A*, *UNC119* |
| [**Nyctalopia**](javascript:void(0);) | *CACNA1F*, *CABP4*, *GNAT1*, *GPR179*, *GRK1*, *GRM6*, *NYX*, *PDE6B*, *RHO*, *SAG*, *SLC24A1*, *TRPM1* |
